# Easy Identification of *Leishmania* Species by Mass Spectrometry

**DOI:** 10.1371/journal.pntd.0002841

**Published:** 2014-06-05

**Authors:** Oussama Mouri, Gloriat Morizot, Gert Van der Auwera, Christophe Ravel, Marie Passet, Nathalie Chartrel, Isabelle Joly, Marc Thellier, Stéphane Jauréguiberry, Eric Caumes, Dominique Mazier, Carine Marinach-Patrice, Pierre Buffet

**Affiliations:** 1 AP-HP, Groupe Hospitalier Pitié-Salpêtrière, Service Parasitologie-Mycologie, Paris, France; 2 Unité d'Immunologie Moléculaire des Parasites, Institut Pasteur de Paris, Paris, France; 3 Department of Biomedical Sciences, Institute of Tropical Medicine, Antwerp, Belgium; 4 Centre National de Référence des Leishmanioses, Montpellier, France; 5 Université Pierre et Marie Curie-Paris6, UMR S945 Paris, France; 6 Institut National de la Santé et de la Recherche Médicale U945, Paris, France; 7 AP-HP, Groupe Hospitalier Pitié-Salpêtrière, Service de Maladie Infectieuse, Paris, France; Swiss Tropical and Public Health Institute, Switzerland

## Abstract

**Background:**

Cutaneous leishmaniasis is caused by several *Leishmania* species that are associated with variable outcomes before and after therapy. Optimal treatment decision is based on an accurate identification of the infecting species but current methods to type *Leishmania* isolates are relatively complex and/or slow. Therefore, the initial treatment decision is generally presumptive, the infecting species being suspected on epidemiological and clinical grounds. A simple method to type cultured isolates would facilitate disease management.

**Methodology:**

We analyzed MALDI-TOF spectra of promastigote pellets from 46 strains cultured in monophasic medium, including 20 short-term cultured isolates from French travelers (19 with CL, 1 with VL). As per routine procedure, clinical isolates were analyzed in parallel with Multilocus Sequence Typing (MLST) at the National Reference Center for *Leishmania*.

**Principal Findings:**

Automatic dendrogram analysis generated a classification of isolates consistent with reference determination of species based on MLST or *hsp70* sequencing. A minute analysis of spectra based on a very simple, database-independent analysis of spectra based on the algorithm showed that the mutually exclusive presence of two pairs of peaks discriminated isolates considered by reference methods to belong either to the *Viannia* or *Leishmania* subgenus, and that within each subgenus presence or absence of a few peaks allowed discrimination to species complexes level.

**Conclusions/Significance:**

Analysis of cultured *Leishmania* isolates using mass spectrometry allows a rapid and simple classification to the species complex level consistent with reference methods, a potentially useful method to guide treatment decision in patients with cutaneous leishmaniasis.

## Author Summary

Cutaneous leishmaniasis is a disease due to a small parasite called *Leishmania*. This parasite causes disfiguring skin lesions that last for months or years. There are many different subtypes of *Leishmania*, each giving rise to lesions of different severity and responding to therapies in its own way. Treating physicians must know as soon as possible which subtype of *Leishmania* is involved to propose the best treatment. Because it is impossible to differentiate the *Leishmania* subtypes microscopically, the identification of the culprit subtype currently requires complex and expensive typing methods, the results of which are generally obtained several weeks after the diagnosis. Here, we have evaluated the ability of a new method using mass spectrometry to differentiate *Leishmania* subtypes. Our results were consistent with those provided by reference typing methods and were obtained rapidly after the parasite had been cultured in vitro. This new method may help physicians know very soon which *Leishmania* subtype is involved thereby facilitating treatment choice.

## Introduction

Cutaneous leishmaniasis (CL) affects 1.5 million patients each year and displays a wide spectrum of clinical forms from small self-resolving papules to severe destructive mucosal lesions. The infecting *Leishmania* species influence the clinical presentation of CL [1] but lesion characteristics are not specific enough for a robust species determination in a given patient [2]–[4].While 2 species of the *Leishmania* subgenus - *L. major* and *L. mexicana* - are associated with frequent spontaneous cure within a few months [3], the 2 main species of the *Viannia* subgenus – *Leishmania braziliensis* and *L. panamensis/guyanensis* are associated with a 1–15% risk of delayed mucosal metastasis [5]. Considering the variable severity of CL, recent guidelines recommend using local therapy whenever possible and systemic therapy if local therapy fails or cannot be performed [3], [6], [7]. This step-wise decision process integrates not only lesion number and size, patients status (age and co-morbidities), but also the infecting species [8].

The influence of the infecting *Leishmania* species on treatment outcome is well established [4], [9], [10]. Thus, species identification is important to determine the clinical prognosis and to select the most appropriate therapeutic regimen. In current clinical practice, treatment decision is generally presumptive, the infecting species being suspected on epidemiological and clinical grounds [3] but this approach requires a specific clinical expertise and frequently updated knowledge of the geographic distribution of *Leishmania* species [3]. A simple, rapid method to type cultured isolates would facilitate an easier and more robust treatment decision based on confirmed species identification.

Available methods to type *Leishmania* in cultured isolates or directly in lesions are still complex and poorly standardized. At present, isolation of the parasite in culture is necessary for identification by multilocus enzyme electrophoresis (MLEE), which has long been the reference for *Leishmania* species identification [11] [12] [13]. Only a few specialized centers currently perform MLEE, the result of which is available several weeks after the isolation of the parasite in culture. These difficulties have led to the development of molecular methods for species identification, generally based on DNA amplification by PCR, followed by single or multilocus sequencing (MLST) or restriction fragment length polymorphism analysis [14] or single strand conformation polymorphism or sequencing of different targets including the *heat shock protein* 70 (*hsp*70) gene [15], [16]. Some of these methods can be applied directly to biological samples avoiding the parasite culture step [17]. However, these molecular methods lack inter-laboratories standardization and require the use of expensive reagents.

Matrix-assisted laser desorption ionization–time-of-flight (MALDI-TOF) mass spectrometry (MS) has emerged as a powerful tool for the identification of microorganisms. Using MALDI-TOF MS, the protein spectral “fingerprint” of an isolate is compared to a reference spectral database, yielding results within 1 hour [18]. Although spectrometers are still relatively expensive, the initial investment is justified by a broad use spanning a wide diversity of microbiological samples [19], and the cost of reagents is very limited. This approach has been applied with success to bacteria, yeasts and filamentous fungi, but to our knowledge, no study on direct identification of protozoans has been published yet [20]–[23]. We have investigated the value of mass spectrometry MALDI-TOF for the identification of *Leishmania* species in patients with CL.

## Materials and Methods

### Medical care and parasite collection

From 2011 through 2013, data and samples were collected each time treatment advice was sought from an expert at our hospital for patients with CL. Diagnosis procedures were not modified by the process, expert treatment advice was part of normal medical care, data and sample collection was in the context of national health surveillance. Patients were informed of the process by their attending physician using a procedure common to all French National Reference Centers (NRC) (http://www.parasitologie.univ-montp1.fr/doc/Declaration_pub_2011.pdf). Data were obtained through the standard reporting form of the NRCL. This form is available online and is anonymous and the anonymisation process is irreversible. The following characteristics are provided on the form: age (children defined as <16 years), sex, clinical form, and for CL or MCL: number of lesions, the presumed place of infection. The collection of parasite isolates was performed in the context of this surveillance program.

### Parasitological confirmation of diagnosis – Species identification

Parasitological diagnosis was performed and analyzed as previously described by lesion scraping, biopsy or aspirate followed by direct examination of Giemsa-stained smears, histological analysis of HES- or Giemsa-stained tissue sections, culture or PCR [24]. To increase the robustness of the analysis, 10 New World isolates were obtained from the Tropical institute in Antwerpen (ITM, Belgium), all strains were re-suspended in 10% glycerol and stored in liquid nitrogen until use.

### Parasite culture

Needle aspirate of skin lesions was performed under local anesthesia then cultured in both Nicolle-McNeal-Novy (NNN) medium and Schneider medium supplemented with 20% fetal calf serum, penicillin and streptomycin [25]. Cultures were kept at 25°C and observed under an inverted microscope in search for motile promastigotes, twice a week for 1 month. Each week positive culture was expanded in 20 mL glass bottles with Schneider medium (20% fetal calf serum, penicillin and streptomycin), one part was frozen in liquid nitrogen −80°, and the other was used for species identification. Aliquots were thawed immediately at 37°, re-suspended in 5 ml of Schneider medium (20% FCS, penicillin streptomycin) and incubated à 27°C. Subcultures were counted daily and analysed at the end of 3-day growth period (growth period being defined à> = ×3 fold multiplication over 3 day), a growth curve was established for each isolate to perform the proteomic analysis during the exponential growth or early stationary phase. For *Leishmania* strains isolated only in NNN medium, promastigotes were concentrated by centrifugation (2500 rpm×10 minutes) and resuspended in 20% HS Schneider medium 24–72 hours before proteomic analysis.

### Molecular identification

Positive cultures were sent to the NRCL for confirmation and species identification using a multilocus sequence typing (MLST) approach based on the analysis of seven single copy coding DNA sequences [26]. Isolates from ITM had already been typed by *hsp* 70 sequencing [15], [16].

### Sample preparation for MALDI-TOF MS

Promastigote suspensions from the expanded cultures were centrifuged 3000 g for 3 minutes and the supernatant removed before the pellet was washed twice in pure water, the pellet was then re-suspended in 300 µL of pure water before adding 900 µL of ethanol. After another round of centrifugation, 10 µL of 70% formic acid and 10 µL pure acetonitrile were added to the residual pellet and the subsequent solution was repeatedly and thoroughly vortexed before a final centrifugation. Each centrifugation step was performed at 10 000 g for 2 min at room temperature.

### MALDI-TOF mass spectrometry

The supernatant was distributed (0.5 µl droplet) in duplicates on a MALDI ground steel sample slide (Bruker-Daltonics, Bremen, Germany) then air-dried. The α-cyano-4-hydroxy-cinnamic acid (CHCA) matrix (Bruker-Daltonics), prepared at a concentration of 50 mg/ml in 50% acetonitrile and 50% water with 2.5% TFA, was sonicated for 5 min before being spotted (0.5 µl) over the dried sample. A DH5-alpha *Escherichia coli* protein extract (Bruker-Daltonics) was deposited on the calibration spot of the sample slide for external calibration. MALDI analysis were performed on a BrukerAutoflex I MALDI TOF mass spectrometer with a nitrogen laser (337 nm) operating in linear mode with delayed extraction (260 ns) at 20 kV accelerating voltage. Each spectrum was automatically collected in the positive ion mode as an average of 500 laser shots (50 laser shots at 10 different spot positions). Laser energy was set just above the threshold for ion production. A mass range between 3,000 and 20,000 m/z (ratio mass/charge) was selected to collect the signals with the AutoXecute tool of flexcontrol acquisition software (Version 2.4; Bruker-Daltonics). Only peaks with a signal/noise ratio >3 were considered. Spectra were eligible for further analysis when the peaks had a resolution better than 600. For each cultivation condition, we collected mass spectra from 2 biological replicates and 4 technical replicates.

### Data processing

Data were processed with Biotyper version 1.1 (Bruker-Daltonics) and ClinProTools 3.0 (bruker-Daltonics) as described [18]. ClinProTools software was used to visualize all spectra as virtual gels and to calculate variability for each of the defined markers. The Biotyper software performs smoothing, normalisation, baseline subtraction, and peak picking using default parameters. Strain comparison was done by principal component analysis (PCA) [27]. Distance values were calculated using Biotyper to build score-oriented dendrograms. Based on these distance values, a dendrogram was generated using the according function of the statistical toolbox of Matlab 7.1 (The MathWorks Inc., USA), which was integrated into Biotyper 1.1. The clustering approach is based on similarity scores implemented in the software.

**Reproducibility** was evaluated by comparing spectra obtained from two independent experiments for each strain. The repeatability and stability of the profiles over generations was tested using a series of extracts obtained from subcultures. One strain was maintained in 2 separate cultures then analyzed in duplicate every 72 h over 5 weeks.

## Results

Of the 46 isolates analyzed, 25 (54%) were from the Old World and 21 (46%) from the New World (Table 1). *L. major* was predominant among Old World isolates (16 isolates/64%), followed by *L. donovani* (3/12%), *L. tropica* (3/12%), *L. infantum (2/8%), and L. killicki* (1/4%). One additionnal isolate from a patient with diffuse cutaneous leishmaniasis acquired in Martinique (French West Indies) related to a trypanosomatid described previously in a very small number of patients [28], [29] was also analyzed. *L. braziliensis/L. peruviana* were predominant among New World isolates (13 isolates/62%), followed by *L. guyanensis/L. panamensis* (5/24%), and *L. mexicana/L. amazonensis* (3/14%). Ten isolates were from ITM in Antwerpen (Belgium), 15 from the Parasitology laboratory at Pitié-Salpêtrière Hospital (Paris, France), 1 from the clinical laboratory at Institut Pasteur (Paris, France). Twenty were collected and analyzed blindly from French travelers (19 with cutaneous leishmaniasis and 1 with visceral leishmaniasis) at the Pitié-Salpêtrière Hospital as part of the routine diagnosis mission of the laboratory between July 2011 and June 2013. A retrospective analysis of our diagnostic activity from 2010 through 2013 showed that 48%, 68%, 82%, 97% of cultures from 47 positive clinical samples were positive after 3, 7, 10, 28 days of incubation, respectively.

**Table 1:** 

Isolate			Patient/disease		MALDI-TOFpeaks													
#	Source	Identification by reference method	Age/Gender	Country of contamination	*“Viannia”* peaks (Da)	*“Leishmania”* peaks (Da)	*L. braziliensis* peaks (Da)		*L. guy./pan. (1)* peaks (Da)		*L. mexicana* peaks (Da)	*L.major* peaks (Da)		*L.killicki* peaks (Da)	*L.tropica* peaks (Da)	*L. don./inf. (2)* peaks (Da)	*L. don.(3)* peaks (Da)	MALDI-TOF identification using algorithm (Fig. 1B)
					7114 & 11121	6153 &7187	5987	6173	6015	6234	5589 & 11180	5630	5937	5726 w/o 7875	5753	7875	5726	
					(±4 & ±7)(3′)	(±3 & ±5)	(±3)	(±3)	(±5)	(±2)	(±3 & ±6)	(±2)	(±2)	(±6 & ±5)	(±3)	(±5)	(±6)	
1	ITM (5)	*L. braziliensis*	NA (4)	Bolivia	X		X	X					X	X		X	X	*L. braziliensis/peruviana*
2	ITM	*L. braziliensis*	NA	Bolivia	X		X	X						X	X	X	X	*L. braziliensis/peruviana*
3	ITM	*L. braziliensis*	NA	Peru	X		X	X						X		X	X	*L. braziliensis/peruviana*
4	ITM	*L. peruviana*	NA	Peru	X		X	X						X		X	X	*L. braziliensis/peruviana*
5	ITM	*L. peruviana*	NA	Peru	X		X	X						X		X	X	*L. braziliensis/peruviana*
6	Clinical (6)	*L. braziliensis*	49/M	Fr. Guy.(7)	X		X	X						X		X	X	*L. braziliensis/peruviana*
7	Clinical	*L. braziliensis*	21/M	Bolivia	X		X	X						X		X	X	*L. braziliensis/peruviana*
8	Clinical	*L. braziliensis*	28/M	Colombia	X		X	X						X	X	X	X	*L. braziliensis/peruviana*
9	Clinical	*L. braziliensis*	49/M	Peru	X		X	X						X		X	X	*L. braziliensis/peruviana*
10	Clinical	*L. braziliensis*	42/M	Brazil	X		X	X						X		X	X	*L. braziliensis/peruviana*
11	Lab (8)	*L. braziliensis*	NA	Brazil	X		X	X						X		X	X	*L. braziliensis/peruviana*
12	Lab	*L. braziliensis*	NA	NA	X		X	X						X		X	X	*L. braziliensis/peruviana*
13	Lab	*L. braziliensis*	NA	NA	X		X	X						X		X	X	*L. braziliensis/peruviana*
14	Clinical	*L. guyanensis*	43/M	Fr. Guy.	X				X	X					X	X	X	*L. guyanensis/panamensis*
15	Clinical	*L. guyanensis*	29/F	NA	X				X	X						X	X	*L. guyanensis/panamensis*
16	Lab	*L. guyanensis*	NA	Costa Rica	X					X						X	X	*L. guyanensis/panamensis*
17	ITM	*L. guyanensis*	NA	Fr. Guy.	X				X	X					X	X	X	*L. guyanensis/panamensis*
18	ITM	*L. panamensis*	NA	Panama	X				X						X	X	X	*L. guyanensis/panamensis*
19	ITM	*L.mex./amazon.(9)*	NA	Brazil		X	X				X							*L. mexicana/amazonensis*
20	ITM	*L. amazonensis*	NA	Peru		X	X				X							*L. mexicana/amazonensis*
21	ITM	*L. mexicana*	NA	Brazil		X	X				X							*L. mexicana/amazonensis*
22	Lab	*L. major*	NA	Morocco		X			X	X		X	X					*L. major*
23	Lab	*L. major*	NA	Tunisia		X			X	X		X	X					*L. major*
24	Clinical	*L. major*	14/F	Mali		X			X	X		X	X					*L. major*
25	Clinical	*L. major*	24/M	Mali		X			X	X		X	X					*L. major*
26	Clinical	*L. major*	21/F	Mali		X			X	X		X	X					*L. major*
27	Lab	*L. major*	62/M	Tunisia		X			X	X		X	X					*L. major*
28	Lab	*L. major*	NA	NA		X			X	X		X	X					*L. major*
29	Lab	*L. major*	72/M	Morocco		X			X	X		X	X					*L. major*
30	Clinical	*L. major*	6/F	Tunisia		X			X	X		X	X					*L. major*
31	Lab	*L. major*	83/M	Algeria		X			X	X		X	X					*L. major*
32	Lab	*L. major*	NA	NA		X			X	X		X	X					*L. major*
33	Lab	*L. major*	NA	NA		X			X	X		X	X					*L. major*
34	Clinical	*L. major*	47/F	Tunisia		X			X	X		X	X					*L. major*
35	Clinical	*L. major*	9/F	Tunisia		X			X	X		X	X					*L. major*
36	Clinical	*L. major*	64/M	Tunisia		X			X	X		X	X					*L. major*
37	Lab	*L. major*	NA	Tunisia		X			X	X		X	X					*L. major*
38	Clinical	*L. tropica*	12/F	Israel		X									X			*L. tropica*
39	Lab	*L. tropica*	NA	Syria		X				X					X			*L. tropica*
40	Clinical	*L. tropica*	67/M	Turkey		X				X					X			*L. tropica*
41	Clinical	*L. trop./kil.(10)*	2/F	Tunisia		X		X	X	X				X			X	*L. killicki*
42	IP Paris (11)	*L. donovani*	NA	NA		X				X						X	X	*L. donovani*
43	Clinical	*L. donovani*	58/F	Turkey		X				X						X	X	*L. donovani*
44	Clinical	*L. donovani*	61/F	Turkey - China		X				X						X	X	*L. donovani*
45	Lab	*L. infantum*	54/M	Turkey - Bulgaria.		X				X						X		*L. infantum*
46	Clinical	*L. infantum*	29/M	France		X				X						X		*L. infantum*

(1) L. guy./pan.: L. guyanensis/panamenesis (4) NA: Information not available (8) Lab: Isolate from the laboratory collection, Pitié-Salpêtrière Hospital (2) L. don./inf.: L. donovani/infantum (5) ITM: Tropical Institute, Antwerpen, Belgium (9) L. mex./amazon.: L.mexicana/amazonensis (3) L. don.: L. donovani (6) Clin: Clinical isolate Pitié-Salpêtrière Hospital, Paris, France (10) L. trop./kil: L. tropica/killicki (3′) Observed variation in peak values in Da (7) Fr. Guy.: French Guyana (11) IP Paris: Isolate from the laboratory collection, Insitut Pasteur Medical Center, Paris, France.

### Process optimization, growth-stage effect and reproducibility

Preliminary tests showed that stable spectra were obtained with 10^6^ promastigotes but that an optimal discrimination of peaks was achieved with 10^7^ promastigotes. Because the culture medium influences spectra (not shown), isolates growing better in NNN medium were transferred for one cycle (i.e., 48–96 hours) in Schneider medium before analysis. The growth kinetics of 2 *L. tropica* isolates was established over two consecutive cycles and spectra were obtained at three stages: exponential (24–72 hours), stationary (72–148 hours) and decay, showing that spectra were reproducible at the exponential and early stationary stages (data not shown). All spectra were then obtained from late exponential/early stationary stages. The reproducibility was further established by analyzing 16 replicates of the same samples for a *L. (L) infantum* and a *L. (V) braziliensis* isolate (**Suppl Fig. S2 & 3**), by analyzing samples from the same isolates of *L. (L) infantum* maintained in culture for several days (**Suppl Fig. S4**), by analyzing samples from the same isolates of *L. (V) braziliensis* analyzed at day 0 and a second culture of the same isolate frozen and thawed for subculture 6 months later (**Suppl Fig. S5**) and by analyzing 3 *L. (L) infantum* and 5 *L. (V) braziliensis* isolates (**Suppl Fig. S6 & 7**). Identification was accurate in all cases. The same approach was also performed with a *L. major* isolate maintained in culture in duplicate for 5 weeks. Spectra lead to the same species identification at all points (not shown).

### Simple, database-independent analysis of spectra: Mutually exclusive presence of 2 pairs of peaks discriminated isolates considered by reference methods as belonging to the *Viannia* or *Leishmania* subgenera

**Figure 1:**
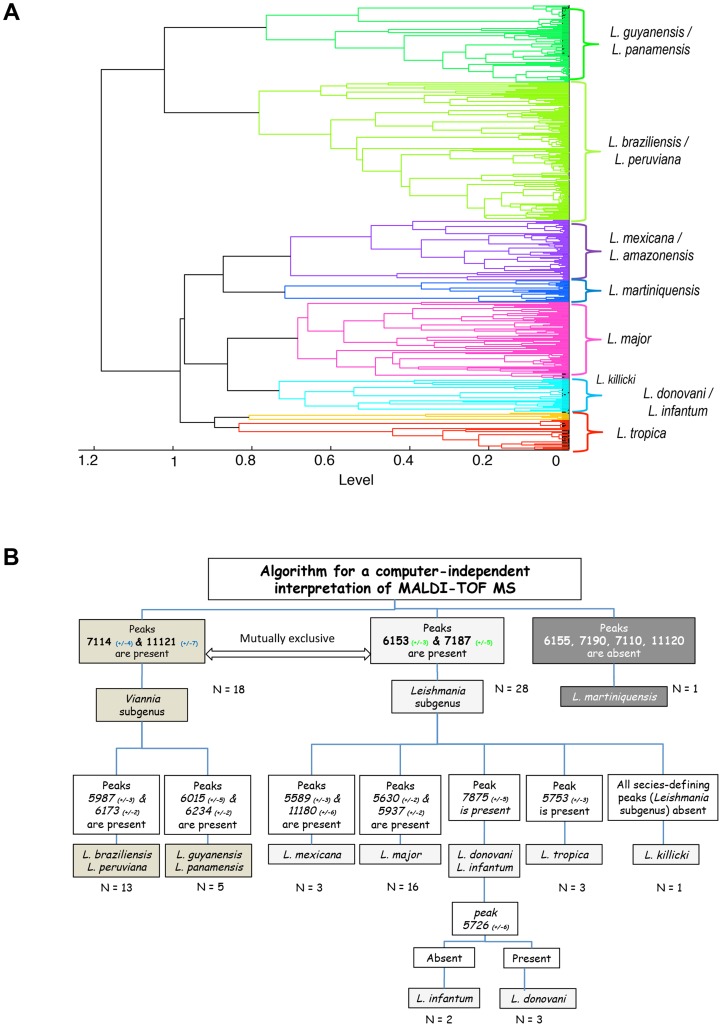
Cluster analysis of MALDI-TOF MS 184 spectra from 46 *Leishmania* isolates (A) with distances displayed in relative units [19], and algorithm for a computer-independent interpretation of MALDI-TOF MS (B) based on presence/absence of peaks as displayed on Table 1.

Peaks 11121 (+/−7) and 7114 (+/−4) were both present in all 18 isolates belonging to the *Viannia* subgenus - 13 *L. braziliensis*, 5 *L. guyanensis/L. panamensis* –and absent in all 28 isolates of the *Leishmania* subgenus - 3 *L.mexicana/L. amazonensis*, 16 *L. major, 5 L. donovani/L. infantum*, 4 *L. tropica/L. killicki (* *Table 1* *,* *Fig. 1* *)*. Conversely, peaks 6153 (+/−3) and 7187 (+/−5) were present in all isolates of the *Leishmania* subgenus and absent in all isolates belonging to the *Viannia* subgenus. Of note, none of these 4 peaks were present in the isolate identified as *L. martiniquensis*. The discriminating power of other peaks was then interpreted in the context of the 2 subgenera.

### Within subgenera, a few peaks allowed classification of isolates down to the species complex level

Within the *Leishmania* subgenus, peaks 5589 (+/−3) and 11180 (+/−6) were present only in *L. mexicana/L. amazonensis* isolates and absent in other isolates, identified by reference methods as *L. killicki, L. tropica, L. major, L. infantum, L. donovani* (Table 1 & Fig. 1B). Similarly, within this *Leishmania* subgenus, peaks 5630 (+/−2) & 5937 (+/−2), or 5753 (+/−3) were present in isolates considered by reference methods as *L. major*, *L. tropica* respectively. Peak 7875 (+/−5) was present in the 5 isolates allocated by MLST to the *L. donovani* complex (*L. donovani & L. infantum*). Peak 5726 (+/−6) was present in the 3 isolates identified as *L. donovani* by reference methods and absent in the 2 *L. infantum* isolates. All those species-defining peaks (*Leishmania* subgenus) were absent in the single *L. killicki* isolate (Table 1). Within the *Viannia* subgenus, the pair of peaks 5987 (+/−3) & 6173 (+/−3) was present in all *L. braziliensis/L. peruviana* isolates and absent in all *L. guyanensis/L. panamensis* isolates. All *L. guyanensis/L. panamensis* isolates expressed either the 6015 (+/−5) or the 6234 (+/2) peak that were both absent in all *L. braziliensis/L. peruviana* isolates. Slight variations for the value of the peaks were observed (Table 1) but – in this relatively limited set of isolates - did not jeopardize the manual, computer independent identification process.

**Figure 2:**
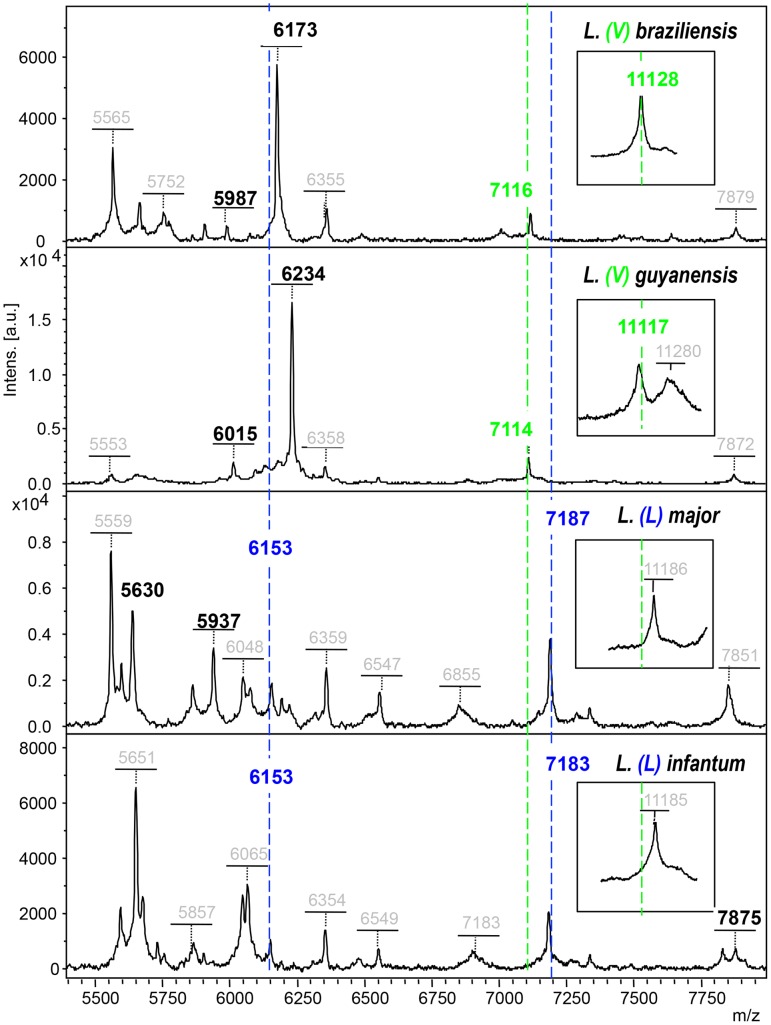
Mass spectra from isolates belonging to either *L. (V) braziliensis, L. (V) guyanensis ((V)* stands for *Viannia* subgenus), *L. (L) major, L. (L) infantum* ((L) stands for *Leishmania* subgenus). The two pairs of peaks discriminating the *Viannia* subgenus from the *Leishmania* subgenus are labeled in green and blue, respectively and indicated by vertical dotted lines. The 11120+/−(7) peak that identifies the *Viannia* subgenus is shown in insert squares at the right side of the figure to improve readability. Peaks differentiating species complexes are labeled with their corresponding molecular weights in colored squares. The software automatically provides the molecular weights for all peaks above signal background (grey labels). Peaks that identify species in each subgenus are shown in Black. A few peaks above background were not labeled on the figure to improve readability, the complete spectra are provided as supplementary **figure S1**.

**Figure 2**: shows mass spectrometry spectra from four different isolates of *Leishmania* included in the reference library (2 from the *Viannia* subgenus 2 from the *Leishmania* subgenus). The four peaks discriminating subgenera and several peaks discriminating species complexes are shown on these spectra, and are labeled with their respective molecular weights thus allowing an easy analysis based on the algorithm (Fig. 1B).

### Automatic dendrogram analysis provided a classification of isolates consistent with determination species by reference methods

A cluster analysis based on a correlation matrix was performed for Old and New world *Leishmania* isolates, in order to assess the ability of the MALDI-TOF MS to generate a classification consistent with that obtained by reference methods. As depicted in (Fig. 1A), the resulting dendrogram for all *Leishmania* isolates showed separate clusters corresponding to the species typed by reference methods, falling appropriately into the 2 subgenera (*Leishmania* and *Viannia*). Isolates considered by MLST as *L. major* were located on one branch, clearly distinguished from isolates considered as *L. donovani/L.infantum* and *L. tropica*. The single *L. killicki* isolate analyzed to date was located in the *Leishmania* subgenus, close to isolates considered as *L. donovani/infantum*. The dendrogram built from PCA differentiated clearly *L. guyanensis*/*L. panamensis* from *L. braziliensis/L. peruviana*s species complexes but segregations between *L. panamensis* and *L. guyanensis*, *L. braziliensis* and *L. peruviana* were not possible at this stage. Isolates belonging to the *L. mexicana complex* fell into the *Leishmania* subgenus on a distinct branch. It appeared close to the single trypanosomatid isolate from the French West Indies (recently named *L. martiniquensis*, Desbois et al., personal data, Fig. 1).

## Discussion

Applied on a set cultured isolates spanning most *Leishmania* species of medical importance, MALDI-TOF MS generated a classification consistent with results obtained by reference methods (MLST or heat-shock protein 70 gene sequencing). This was achieved using a simple, database independent analysis of MALDI-TOF spectra based on the algorithm. The simplicity of the analytical procedure allowed a minute output of results as soon as fair parasite growth was obtained in monophasic medium. Taken together, these observations suggest that MALDI-TOF may be a useful tool to facilitate treatment decision in cutaneous leishmaniasis.

Treatment of CL should indeed be based on species identification [1], [3], [4], [9], [10], [30]–[32]. For example, systemic antimony is generally more effective in patients infected with *L. braziliensis* than in patients infected with *L. guyanensis* [33], [34] or *L. mexicana* [9]. Conversely pentamidine is frequently used to treat *L. guyanensis/panamensis* CL [35] but is poorly effective in *L. braziliensis* CL [36]. Many patients get infected in places where both species circulate and may therefore receive initially first course of a suboptimal treatment. In the Old World, *L. major* can be treated effectively and easily with a 3^rd^ generation aminoglycoside ointment [37], [38] but the efficacy of this topical formulation in patients infected with *L. tropica* or *L. infantum* is as yet unknown [4]. In all these situations, rapid species identification should help adopt the most appropriate option in a majority of patients. Because more than 80% of cultures in our context are positive in the first 10 days after sampling, and because it takes a few hours to obtain the MALDI-TOF spectrum, in many instances time-to-identification is now short enough to with-hold treatment decision until species identification is available. Analysis of a higher number of isolates will be necessary to deliver a more solid dendrogram, particularly to determine whether MALDI-TOF MS can achieve a robust discrimination between *L. braziliensis* and *L. peruviana*, *L. panamensis* and *L. guyanensis*, *L. donovani* and *L. infantum*, *L. mexicana* and *L. amazonensis*. Fortunately, in current algorithms therapeutic decision in cutaneous leishmaniasis is not heavily impacted by these differentials [3], [6].

The approach presented here has no taxonomic ambition but was evaluated for potential use in medical practice. We limit our conclusions to the ability of MALDI-TOF MS to generate clusters congruent with those raised by reference methods. Because *Leishmania* species determination is complex, extension of explorations will be performed in the context of multinational networks such as the LeishMan consortium (http://www.leishman.eu) that merges information from several European countries and benefits from the presence of experts with strong expertise in *Leishmania* species identification [14]–[16]. Interpretation of complex spectra in very rare cases of co-infection with two *Leishmania* species will be attempted in this context. Optimization of pre-analytical steps, including culture conditions, parasite concentration in pellets and protein extraction was important for a robust interpretation of spectra. We selected a 72–96 h incubation period, corresponding to the exponential phase or early stationary phase of growth to limit variations in protein content. Schneider, an axenic medium (20% fetal calf serum, penicillin and streptomycin), was chosen as the reference because its supports rapid growth of most isolates and is associated with reproducible spectra. The need for a culture step is a weakness of mass spectrometry shared with several other typing methods. In rare instances, parasite isolation was difficult or slow. We partially circumvented this bottleneck by culturing clinical samples simultaneously on Schneider and NNN medium. Once adapted, NNN-dependent isolates were transferred for one cycle in Schneider medium then processed for MALDI-TOF analysis. In the long-term, the approach may be further simplified by using dipsticks developed from discriminating peaks. Sensitive protein detection using immunochromatography directly from a lesion scraping or aspirate – as currently developed for diagnosis- may indeed by-pass the culture step and further accelerate species identification.

Another relative limitation of mass spectrometry is that the method is currently handled by reference centers only. However, because mass spectrometers have a wide spectrum of medical applications in microbiology, prices are dropping and cheaper versions are emerging.

In the short term, we found that MALDI-TOF (MS) was a promising approach to generate spectra from *Leishmania* promastigotes with high identification at the species level consistent with the reference method. A limitation of the technique is the need for cultivation parasites. Nevertheless, as compared with molecular biology [39], this approach offers great advantages, in particular speed, simplicity, cost for isolate identification and was easy to integrate into the organization of a clinical laboratory. Not least, the intuitive interpretation of spectra was well-suited for allowing for close interactions between parasitologists and clinicians. These strengths should predictably facilitate rapid treatment decision in cutaneous leishmaniasis.

## Supporting Information

Figure S1 **Mass spectra from isolates belonging to either** ***L. (V) braziliensis, L. (V) guyanensis ((V)*** **stands for** ***Viannia*** **subgenus),** ***L. (L) major, L. (L) infantum*** **((L) stands for** ***Leishmania*** **subgenus).** The two pairs of peaks discriminating the *Viannia* subgenus from the *Leishmania* subgenus are labeled in green and blue, respectively and indicated by vertical dotted lines. Peaks differentiating species complexes are labeled with their corresponding molecular weights. The software automatically provides the molecular weights for all peaks above signal background (grey labels). Peaks that identify species in each subgenus are shown in Black.
(TIF)

Figure S2 **Virtual gels (based on Mass spectra) and representative mass spectra from a L. (L)** ***infantum*** **isolate (see** **table 1** **for origin).** The two peaks discriminating the *Leishmania* subgenus (6153+/−3, and 7187+/−5) are labelled in blue. *L. (L) infantum*-identifying peak (7875+/−5) is shown in black with labeled molecular weight. Virtual gels and a representative spectrum from sixteen analyses corresponding to a single *Leishmania (L) infantum* isolate.
(TIF)

Figure S3 **Virtual gels (based on Mass spectra) and representative mass spectra from a** ***L. (V) braziliensis*** **isolate (see** **table 1** **for origin).** The two peaks discriminating the *Viannia* subgenus (7114+/−4, and 11121+/−7) are labelled in green. The *L. (V) braziliensis* -identifying peaks (5987+/−3, and 6173+/−3) are labelled in black with their respective molecular weights. Virtual gels and a representative spectrum from sixteen analyses corresponding to a single *Leishmania (V) braziliensis* isolate.
(TIF)

Figure S4 **Virtual gels (based on Mass spectra) and representative mass spectra from a L. (L)** ***infantum*** **isolate (see** **table 1** **for origin).** The two peaks discriminating the *Leishmania* subgenus (6153+/−3, and 7187+/−5) are labelled in blue. *L. (L) infantum*-identifying peak (7875+/−5) is shown in black with labeled molecular weight. Virtual gels and a representative spectrum from 4 samples from a *L. (L) infantum* culture analyzed at day 0, day1, day3 and day7. In 2 occasions no signal was detected from the sample (*).
(TIF)

Figure S5 **Virtual gels (based on Mass spectra) and representative mass spectra from a** ***L. (V) braziliensis*** **isolate (see** **table 1** **for origin).** The two peaks discriminating the *Viannia* subgenus (7114+/−4, and 11121+/−7) are labelled in green. The *L. (V) braziliensis* -identifying peaks (5987+/−3, and 6173+/−3) are labelled in black with their respective molecular weights. Virtual gels and a representative spectrum from 3 samples from a first culture analyzed at day 0 and a second culture analyzed twice of the same *Leishmania (V) braziliensis* isolate frozen and thawed for subculture 6 months later.
(TIF)

Figure S6 **Virtual gels (based on Mass spectra) and representative mass spectra from a L. (L)** ***infantum*** **isolates (see** **table 1** **for origin).** The two peaks discriminating the *Leishmania* subgenus (6153+/−3, and 7187+/−5) are labelled in blue. *L. (L) infantum*-identifying peak (7875+/−5) is shown in black with labeled molecular weight. Virtual gels and a representative spectrum from 3 different strains of *L. (L) infantum*.
(TIF)

Figure S7 **Virtual gels (based on Mass spectra) and representative mass spectra from a** ***L. (V) braziliensis*** **isolates (see** **table 1** **for origin).** The two peaks discriminating the *Viannia* subgenus (7114+/−4, and 11121+/−7) are labelled in green. The *L. (V) braziliensis* -identifying peaks (5987+/−3, and 6173+/−3) are labelled in black with their respective molecular weights. Virtual gels and a representative spectrum from 5 different strains of *L. (V) braziliensis*.
(TIF)
