# Supplementary figures and images for: Easy Identification of Leishmania Species by Mass Spectrometry
Source: PLoS Negl Trop Dis. 2014 Jun 5;8(6):e2841. doi: 10.1371/journal.pntd.0002841 (PMC4046964; doi:10.1371/journal.pntd.0002841)

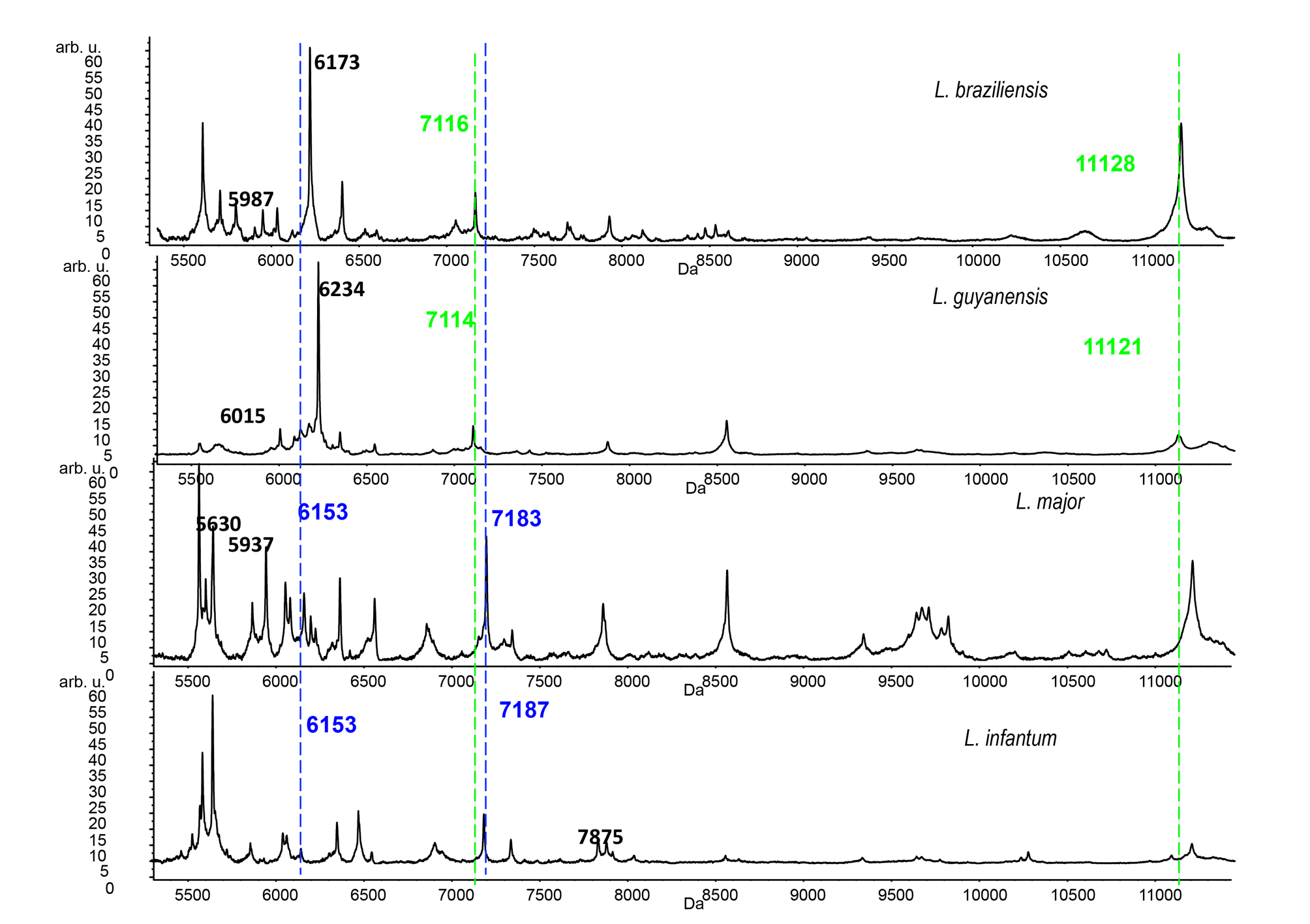

Supplement: Figure S1 — Mass spectra from isolates belonging to either L. (V) braziliensis, L. (V) guyanensis ((V) stands for Viannia subgenus), L. (L) major, L. (L) infantum ((L) stands for Leishmania subgenus). The two pairs of peaks discriminating the Viannia subgenus from the Leishmania subgenus are labeled in green and blue, respectively and indicated by vertical dotted lines. Peaks differentiating species complexes are labeled with their corresponding molecular weights. The software automatically provides the molecular weights for all peaks above signal background (grey labels). Peaks that identify species in each subgenus are shown in Black. (TIF) [file pntd.0002841.s001.tif]

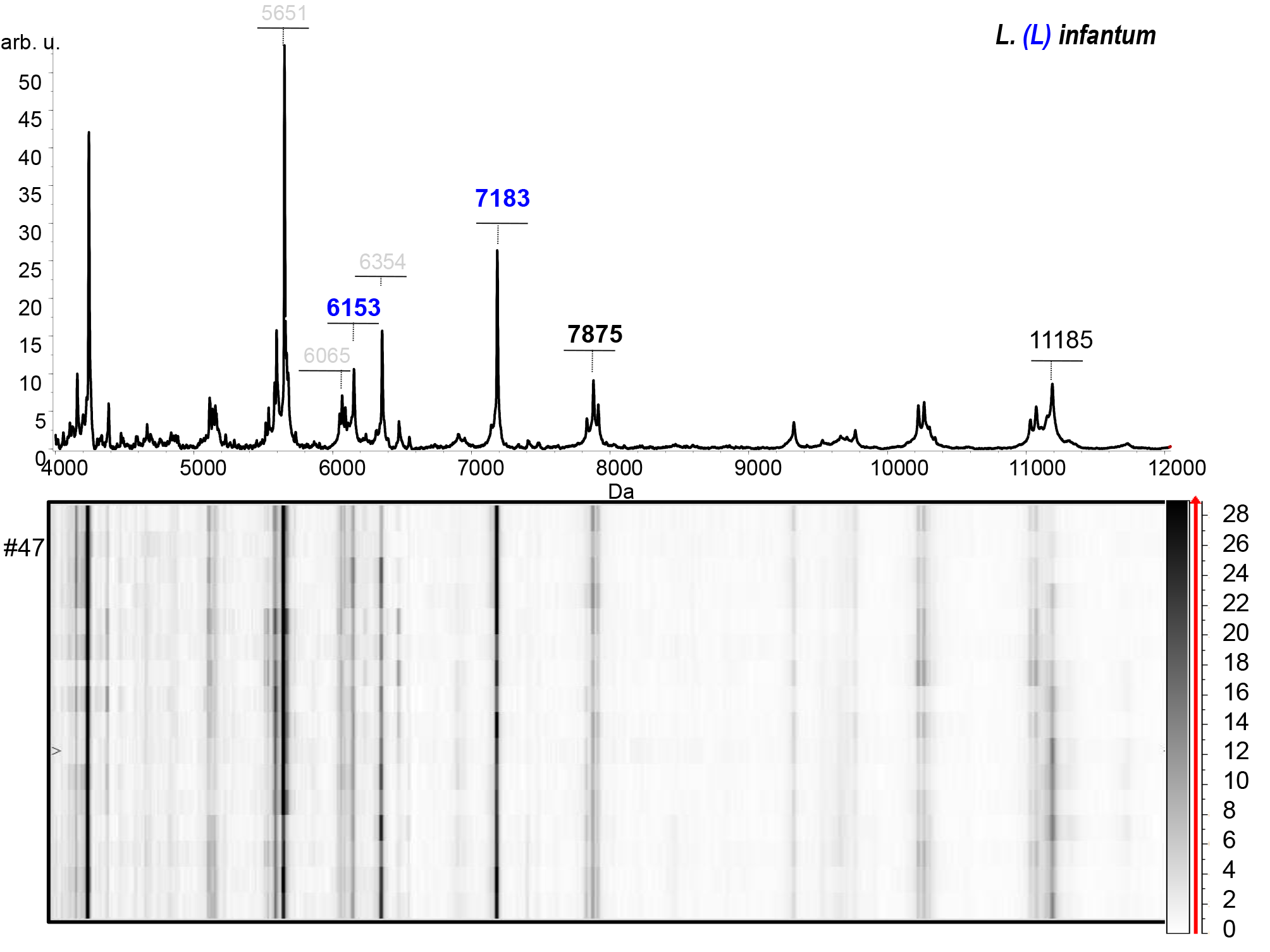

Supplement: Figure S2 — Virtual gels (based on Mass spectra) and representative mass spectra from a L. (L) infantum isolate (see table 1 for origin). The two peaks discriminating the Leishmania subgenus (6153+/−3, and 7187+/−5) are labelled in blue. L. (L) infantum-identifying peak (7875+/−5) is shown in black with labeled molecular weight. Virtual gels and a representative spectrum from sixteen analyses corresponding to a single Leishmania (L) infantum isolate. (TIF) [file pntd.0002841.s002.tif]

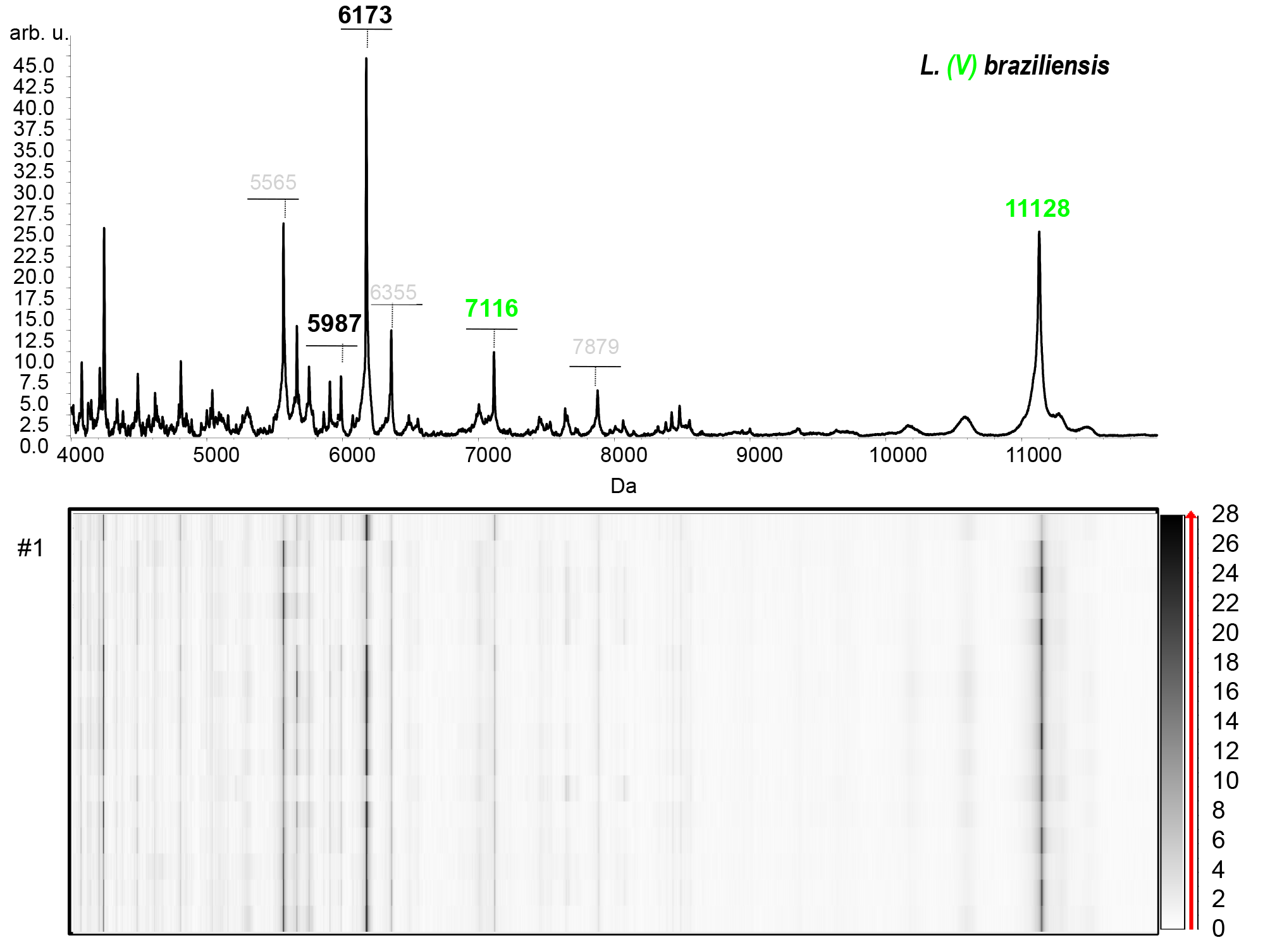

Supplement: Figure S3 — Virtual gels (based on Mass spectra) and representative mass spectra from a L. (V) braziliensis isolate (see table 1 for origin). The two peaks discriminating the Viannia subgenus (7114+/−4, and 11121+/−7) are labelled in green. The L. (V) braziliensis -identifying peaks (5987+/−3, and 6173+/−3) are labelled in black with their respective molecular weights. Virtual gels and a representative spectrum from sixteen analyses corresponding to a single Leishmania (V) braziliensis isolate. (TIF) [file pntd.0002841.s003.tif]

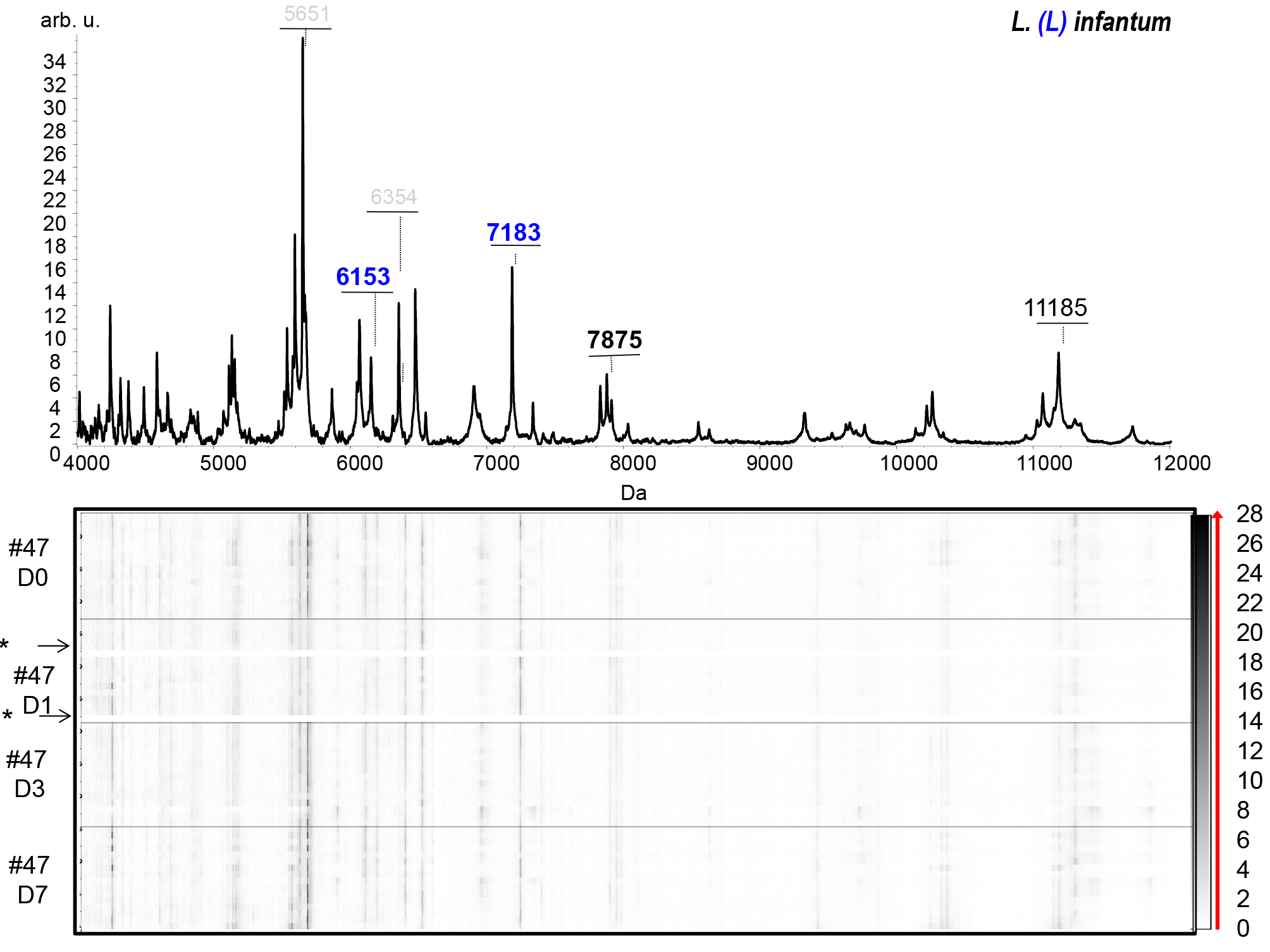

Supplement: Figure S4 — Virtual gels (based on Mass spectra) and representative mass spectra from a L. (L) infantum isolate (see table 1 for origin). The two peaks discriminating the Leishmania subgenus (6153+/−3, and 7187+/−5) are labelled in blue. L. (L) infantum-identifying peak (7875+/−5) is shown in black with labeled molecular weight. Virtual gels and a representative spectrum from 4 samples from a L. (L) infantum culture analyzed at day 0, day1, day3 and day7. In 2 occasions no signal was detected from the sample (*). (TIF) [file pntd.0002841.s004.tif]

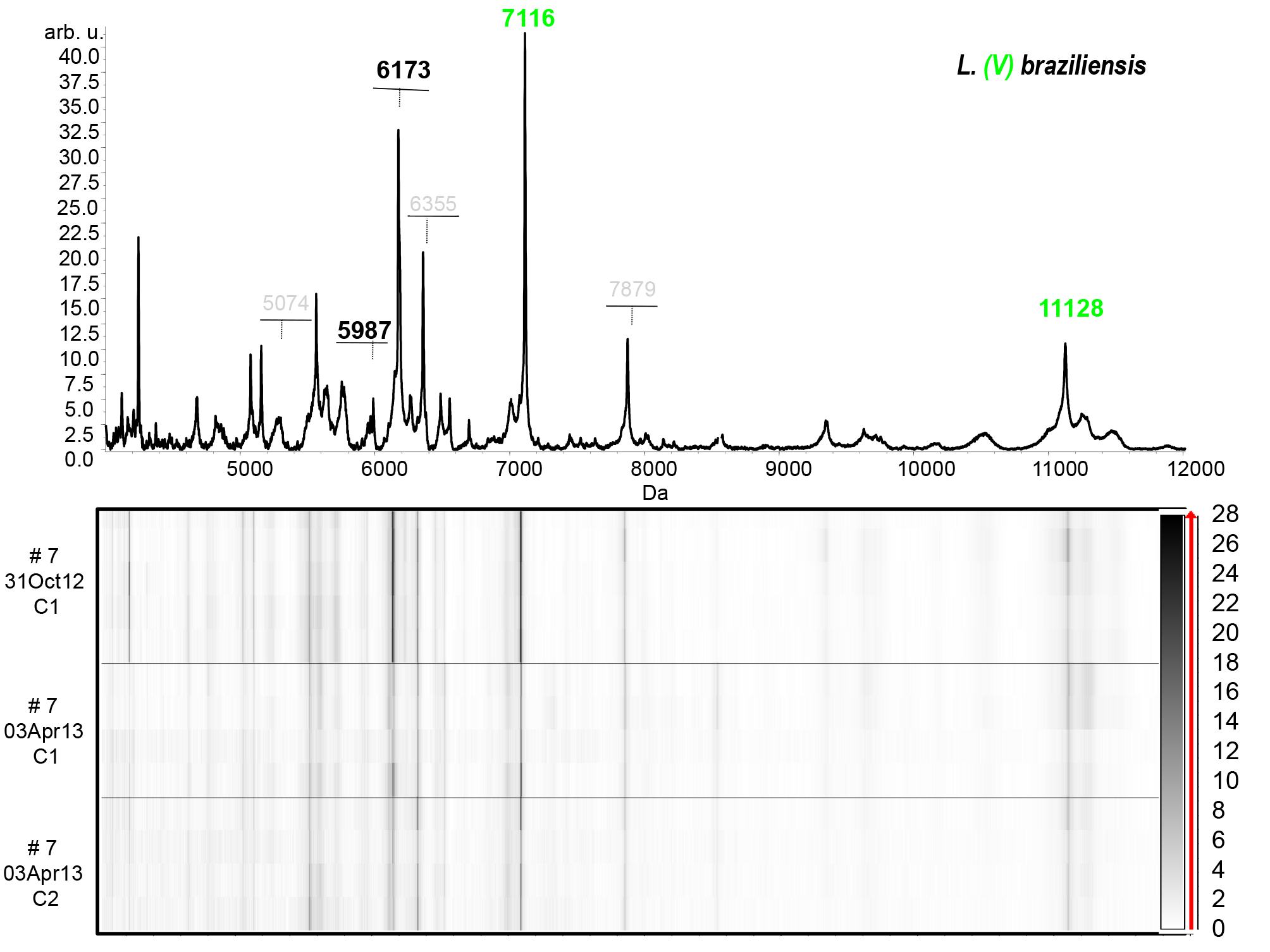

Supplement: Figure S5 — Virtual gels (based on Mass spectra) and representative mass spectra from a L. (V) braziliensis isolate (see table 1 for origin). The two peaks discriminating the Viannia subgenus (7114+/−4, and 11121+/−7) are labelled in green. The L. (V) braziliensis -identifying peaks (5987+/−3, and 6173+/−3) are labelled in black with their respective molecular weights. Virtual gels and a representative spectrum from 3 samples from a first culture analyzed at day 0 and a second culture analyzed twice of the same Leishmania (V) braziliensis isolate frozen and thawed for subculture 6 months later. (TIF) [file pntd.0002841.s005.tif]

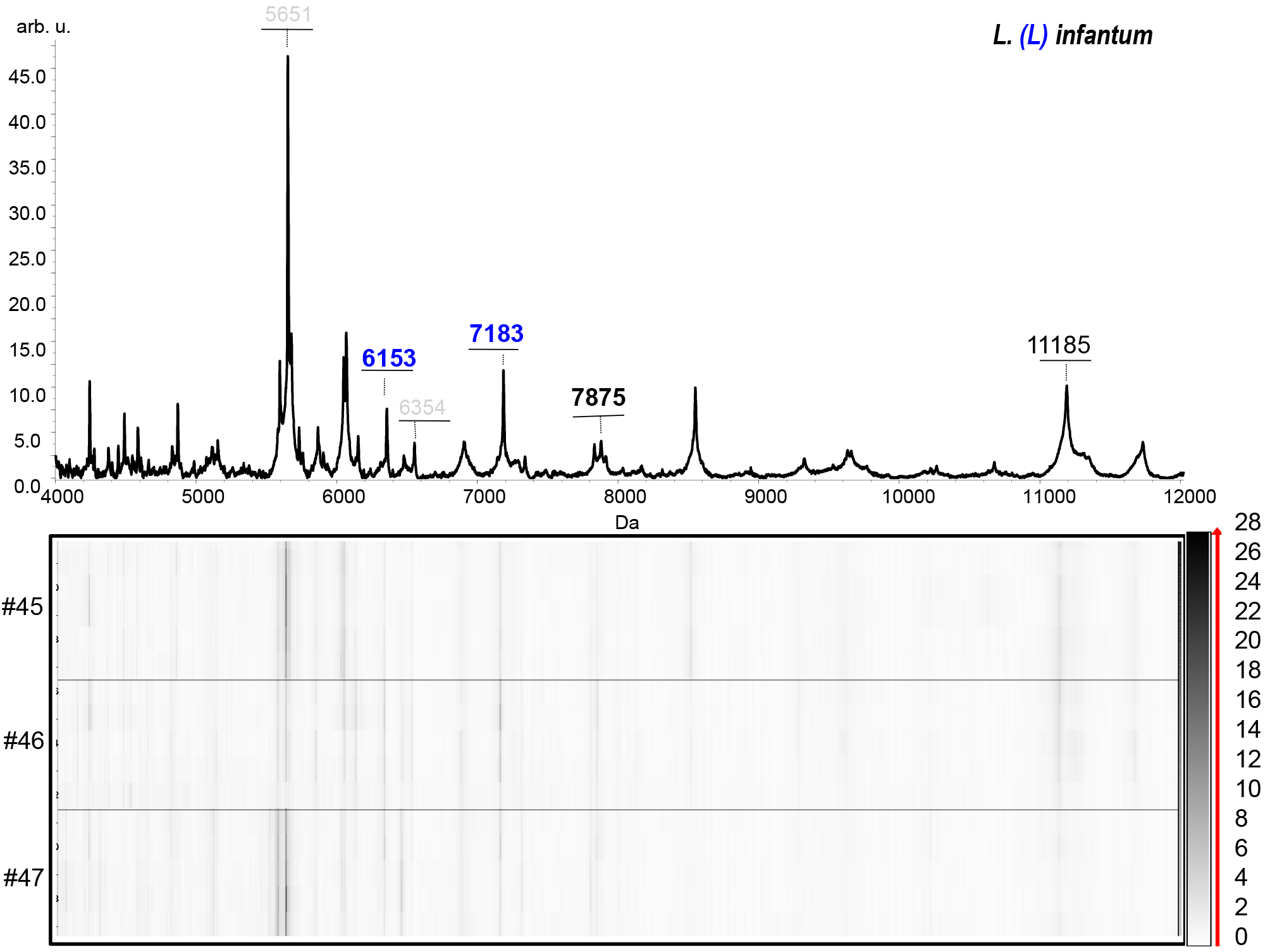

Supplement: Figure S6 — Virtual gels (based on Mass spectra) and representative mass spectra from a L. (L) infantum isolates (see table 1 for origin). The two peaks discriminating the Leishmania subgenus (6153+/−3, and 7187+/−5) are labelled in blue. L. (L) infantum-identifying peak (7875+/−5) is shown in black with labeled molecular weight. Virtual gels and a representative spectrum from 3 different strains of L. (L) infantum. (TIF) [file pntd.0002841.s006.tif]

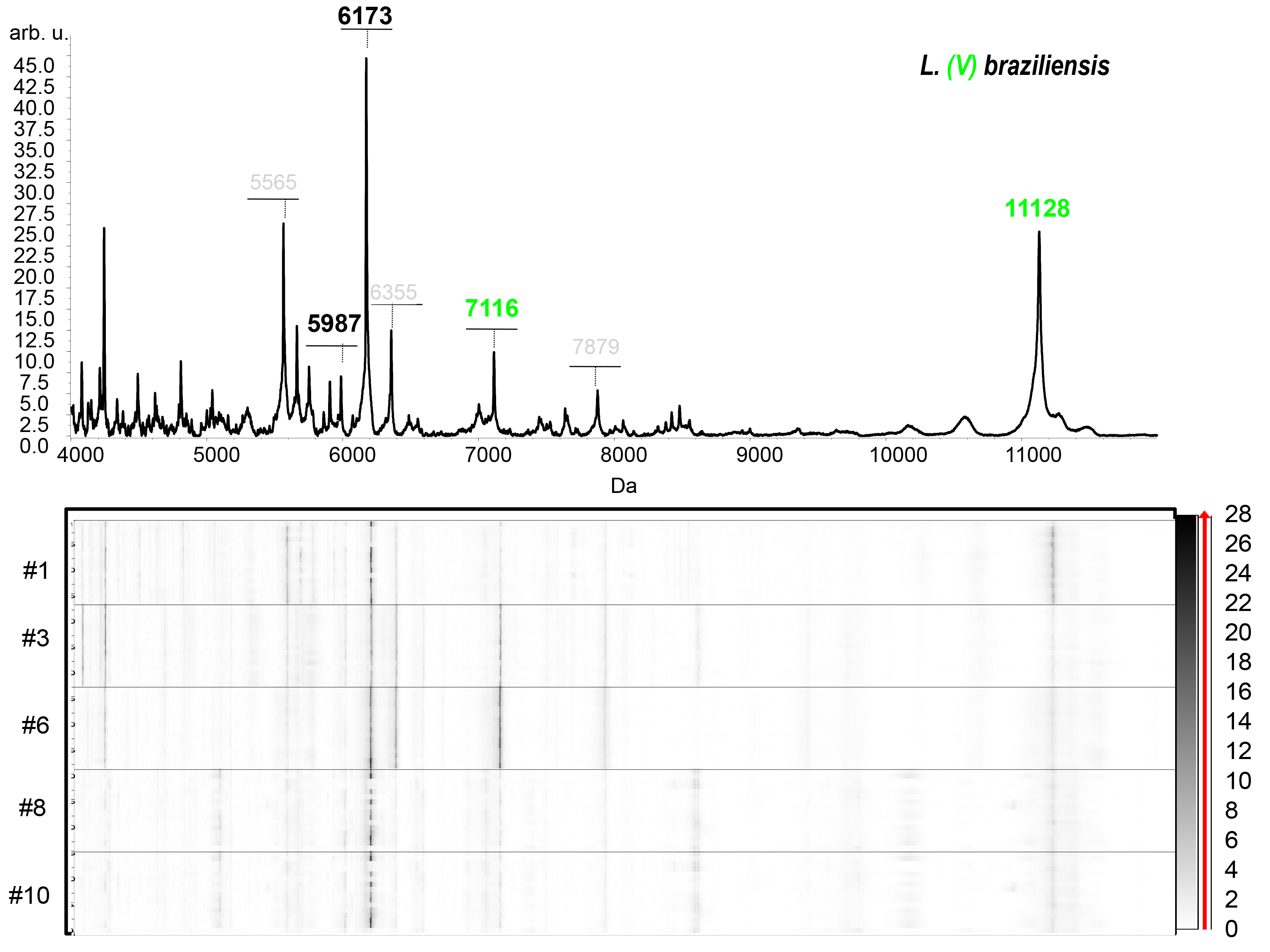

Supplement: Figure S7 — Virtual gels (based on Mass spectra) and representative mass spectra from a L. (V) braziliensis isolates (see table 1 for origin). The two peaks discriminating the Viannia subgenus (7114+/−4, and 11121+/−7) are labelled in green. The L. (V) braziliensis -identifying peaks (5987+/−3, and 6173+/−3) are labelled in black with their respective molecular weights. Virtual gels and a representative spectrum from 5 different strains of L. (V) braziliensis. (TIF) [file pntd.0002841.s007.tif]
